# Pregnancy Pause: Extreme Heat Linked to Shortened Gestation

**DOI:** 10.1289/ehp.119-a443b

**Published:** 2011-10-01

**Authors:** Tanya Tillett

**Affiliations:** Tanya Tillett, MA, of Durham, NC, is a staff writer/editor for *EHP*. She has been on the *EHP* staff since 2000 and has represented the journal at national and international conferences.

Pregnancy tends to make women more vulnerable to heat stress. Added fat deposits and the attendant decrease in the ratio of body surface area to body mass mean a woman’s body is less able to cool off by losing heat to the environment. Heat stress has been linked in earlier studies to induction of uterine contractions, increased secretion of the childbirth-related hormones oxytocin and prostaglandin F_^2〈^_, and increased levels of heat-shock protein 70 (which has been linked to preterm delivery). A new study now suggests maternal exposure to extreme heat may have an immediate effect on pregnancy duration [*EHP* 119(10):1449–1453; Dadvand et al.].

The Spanish-based research team analyzed birth data for 7,585 women who delivered at the Hospital Clinic of Barcelona between January 2001 and June 2005. They used national data on daily heat and temperature to calculate which days during that period exceeded the 90th (HI_90_), 95th (HI_95_), and 99th (HI_99_) percentiles for heat index for the longer period of 1983–2006.

Pregnancies ranged from 22.2 to 43.5 weeks, with an average of 40 weeks. The results showed that all three HI percentiles were associated with a reduction in pregnancy duration. An HI_90_ episode on the day before delivery (lag 1) was associated with a 1-day reduction in average pregnancy duration, a lag 1 HI_95_ episode was associated with a 2-day reduction, and a lag 1 HI_99_ event was connected to a 5-day reduction. There was little evidence of an association beyond the delivery date and the day before, suggesting that any effect heat stress may have on pregnancy duration is immediate.

When the investigators assessed Europeans versus non-Europeans, they found a relationship between an HI_99_ episode on the day of delivery and an 8-day reduction in average pregnancy duration for European women compared with a 1-day reduction for non-Europeans.

The study was limited by the inability to control for air-conditioner use, study the associations in ethnic groups besides Europeans, or examine how the length of extreme heat episodes affected pregnancy duration. With future climate projections including increases in the frequency and intensity of extreme heat conditions—and given that a reduction of even a week in the length of pregnancy has been linked to adverse health outcomes in newborns—the authors contend that future studies should consider these factors to help inform appropriate public health interventions.

